# Risk factors affecting patients survival with colorectal cancer in Morocco: survival analysis using an interpretable machine learning approach

**DOI:** 10.1038/s41598-024-51304-3

**Published:** 2024-02-12

**Authors:** Imad El Badisy, Zineb BenBrahim, Mohamed Khalis, Soukaina Elansari, Youssef ElHitmi, Fouad Abbass, Nawfal Mellas, Karima EL Rhazi

**Affiliations:** 1 Mohammed VI Center for Research and Innovation, Rabat, Morocco; 2 International School of Public Health, Mohammed VI University of Sciences and Health, Casablanca, Morocco; 3 INSERM, IRD, SESSTIM, Sciences Economiques & Sociales de la Santé & Traitement de l’Information Médicale, Aix Marseille Univ, Marseille, France; 4 Faculty of Medicine, Pharmacy & Dental Medicine, Sidi Mohamed Ben Abdillah University, Fez, Morocco; 5 Department of Oncology, University Hospital Hassan II, Sidi Mohamed Ben Abdellah University, Fez, Morocco; 6 Higher Institute of Nursing Professions and Technical Health, Rabat, Morocco; 7 Laboratory of Biostatistics, Clinical, and Epidemiological Research, Faculty of Medicine and Pharmacy, Department of Public Health, Mohamed V University, Rabat, Morocco; 8 Laboratory of Epidemiology and Research in Health Sciences, Department of Epidemiology and Public Health, Faculty of Medicine of Fez, Sidi Mohamed Ben Abdillah University, Fez, Morocco

**Keywords:** Oncology, Risk factors

## Abstract

The aim of our study was to assess the overall survival rates for colorectal cancer at 3 years and to identify associated strong prognostic factors among patients in Morocco through an interpretable machine learning approach. This approach is based on a fully non-parametric survival random forest (RSF), incorporating variable importance and partial dependence effects. The data was povided from a retrospective study of 343 patients diagnosed and followed at Hassan II University Hospital. Covariate selection was performed using the variable importance based on permutation and partial dependence plots were displayed to explore in depth the relationship between the estimated partial effect of a given predictor and survival rates. The predictive performance was measured by two metrics, the Concordance Index (C-index) and the Brier Score (BS). Overall survival rates at 1, 2 and 3 years were, respectively, 87% (SE = 0.02; CI-95% 0.84–0.91), 77% (SE = 0.02; CI-95% 0.73–0.82) and 60% (SE = 0.03; CI-95% 0.54–0.66). In the Cox model after adjustment for all covariates, sex, tumor differentiation had no significant effect on prognosis, but rather tumor site had a significant effect. The variable importance obtained from RSF strengthens that surgery, stage, insurance, residency, and age were the most important prognostic factors. The discriminative capacity of the Cox PH and RSF was, respectively, 0.771 and 0.798 for the C-index while the accuracy of the Cox PH and RSF was, respectively, 0.257 and 0.207 for the BS. This shows that RSF had both better discriminative capacity and predictive accuracy. Our results show that patients who are older than 70, living in rural areas, without health insurance, at a distant stage and who have not had surgery constitute a subgroup of patients with poor prognosis.

## Introduction

Colorectal cancer (CRC) is the 3rd most diagnosed cancer in the world, with more than 1.931 million new cases in 2020 worldwide and 4558 new cases in Morocco, representing almost 7.7 percent of all new cancer cases in the country^1^. Several factors are significantly associated with the prognosis of CRC patients^2–4^. Whether these factors are related to patient characteristics, treatments, or even the healthcare system in general, it is essential to study their effects in order to develop a care strategy adapted to the local context.

Moreover, CRC imposes an economic burden that translates into direct medical expenses (cost of screening, hospitalization, treatment, transportation, etc.) and indirect expenses such as loss of productivity^5–7^.

Financial and insurance barriers have a major effect on the survival of patients with cancer. They are generally identified as directly related to health care utilization. For example, financial concerns have been found to prevent uninsured people from seeking care unless they were in severe pain or thought they were going to die^8,9^. In a study on CRC patients, 18% reported untreated rectal bleeding and 20% reported a change in bowel habit but never sought medical attention. The main reason was that they did not consider this symptom to be serious^10^.

Furthermore, there is considerable epidemiological and observational evidence that the risk of CRC is closely related to lifestyle, particularly diet and physical activity^11–13^.

CRC, being one of the leading causes of cancer-related deaths worldwide, presents intricate challenges to researchers and clinicians alike. Its multifaceted nature is evident in its diverse etiology, varied genetic mutations, and the influence of both internal and environmental factors on its progression. Patients with seemingly similar clinical presentations can have starkly different outcomes, making prognosis and treatment a challenging endeavor. In addition, the interplay between genetic factors, lifestyle choices, environmental exposures, and gut microbiota adds layers of complexity to the disease's understanding^14^. Conventional epidemiological methodologies frequently face challenges in effectively disentangling the intricate and interdependent factors involved in disease etiology. Therefore, there's a pressing need for sophisticated analytical tools that can sift through this complexity, discern patterns, and provide actionable insights to improve patient outcomes.

Advancements in precision medicine will necessitate more personalized prognostic evaluations for patients to guide appropriate treatments. Traditional statistical techniques for creating prognostic models involve substantial human input, encompassing the selection of prognostic factors based on disease understanding, variable manipulation and filling, and validating model assumptions^15^.

In this sense, machine learning provides a promising alternative to conventional statistical models, in identifying prognostic factors within vast and intricate datasets. Contrary to the standard approach of pre-selecting prognostic factors grounded in disease understanding, machine learning employs a non-deterministic strategy, letting the data itself uncover pivotal characteristics essential for precise predictions. This evolution from a pre-determined selection to a data-driven exploration signifies a substantial shift in predictive and prognostic modeling^16,17^.

Specifically, machine learning methods include automatic variable selection and non-parametric modeling strategies. These techniques can adeptly manage a multitude of predictors while making fewer presumptions about the relationships between particular variables and the desired outcomes. Consequently, such methodologies potentially reduce the extent of human input needed in crafting prognostic models^18^.

For instance, random survival forests can accommodate nonlinearities and interactions among variables. They are not confined to a uniform baseline hazard for all patients, circumventing the inherent assumption in Cox proportional hazards models^19^. Machine learning techniques can also make use of more data, which in different settings may give rise to further improved performance over conventional models. Also, variable selection allows its use in contexts where risk factors are unknown^20^.

Futheremore, in the survival analysis framework, parametric models rest upon assumptions that might not be upheld in real-world scenarios. The semi-parametric Cox proportional hazards models (Cox PH), for example, operate under the assumption that changes in predictor variables lead to a multiplicative effect on the baseline hazard during the period of observation^21^.

Additionally, addressing missing data requires a distinct approach, such as multiple imputation. Many imputation methods assume that missing data occur randomly (missing at random). While this assumption holds in many clinical settings, it's not always guaranteed, especially in prognostic studies^22^.

In an optimal research setting, a study based on the entire population would be the preferred approach, providing a broad range of insights. However, due to limited resources and the lack of a nationwide information system, using data from hospitals becomes necessary. While population-based data offers a lot of information, hospital data has its own unique characteristics. Hospital datasets contain a variety of details, including clinical information, biomarkers, and healthcare expenses^23^.

This research focuses on a specific situation where various factors interact and affect the prognostic of CRC patients. The main goal of this study was to carefully explore the factors that shaped the risks, prognosis, and survival of CRC, particularly within the healthcare system of Morocco. As this investigation delves into the complex set of factors related to CRC and its various causes, its importance goes beyond just academic research. The significance of the study lies on its potential to provide practical insights that can guide specific healthcare policies and actions, thus filling gaps in healthcare delivery and improving patient outcomes.The aim of our study was to assess the overall survival rates for CRC at 3 years and to identify associated strong prognostic factors among patients in Morocco through an interpretable machine learning approach based on a fully non-parametric survival random forest with variable importance and partial dependence effects.

## Methods

### Study design and data collection

We performed a retrospective analysis of 343 patients diagnosed and followed at Hassan II University Hospital. The date of diagnosis of CRC indicated the start of the observation period. Patients were followed from January 2009 to January 2015 until death or censored at the end of the study. The end-point was set at 36 months from the date of diagnosis. All included patients were incident cases, whose dates of diagnoses occur within the active study period, ensuring that potential biases associated with disease duration prior to study entry were minimized. This approach, focusing exclusively on incident cases, effectively reduces the risk of biases related to left truncation censoring.

Data about patients’ characteristics was extracted from the patients’ medical records and supplemented with an active follow-up to record vital status and observed survival times. Eligible patients had a histologically confirmed diagnosis of CRC. Patients with histological types other than adenocarcinoma (N = 200) and diffuse lattice type (N = 50) were excluded. Similarly, patients with uninformed medical records were removed (N = 150).

For each patient, information on sex, age, insurance, residency, delay to treatment, personal history, tumor site, stage, tumor differentiation, histological type, surgery, and MSI/MSS status, was extracted.

### Ethical considerations

This study, conducted under the strict observance of relevant guidelines and regulations, was retrospectively designed and only involved the collection of medical history data. Due to this retrospective nature, the requirement for informed consent was waived by the Local Ethics Committee of the Hassan II University Hospital of Fes. The approval for this study was granted by the aforementioned Ethics Committee, as attested by the reference number 05/18.

### Statistical analysis

A descriptive analysis of the study sample was conducted.

While multiple imputation remains as the gold standard in prognostic studies, its utilization is incompatible with machine learning algorithms. Specifically, multiple imputation requires the generation of a distribution for the missing values, and the subsequent estimation of multiple models of the missing data, with the results pooled using Rubin's rules. This process is feasable and attainable with regression models, as they yield coefficients of estimation, allowing the pooled coefficients and their standard errors to incorporate the uncertainty surrounding the missing data imputation.

However, since the majority of machine learning algorithms take a single dataset as input, the pooling procedure becomes challenging to implement. Confronted with this limitation, we adopted a method of single imputation. Single imputation of missing data was done using the missRanger algorithm. It implements an imputation approach based on the random forest algorithm combined with the predictive mean matching method^24,25^. This is a non-parametric imputation method that makes no prior assumptions about the distribution of the data. It directly predicts missing values using a random forest trained on the observed parts of the dataset. The imputation is performed iteratively until a convergence criterion is reached.

Before settling on our final model based on single imputation, a comparison was conducted among the complete cases, multiple imputation relying on Random Forest (mice RF) with 10 datasets, and single imputation based on Random Forest (missRanger).

Overall survival rates (1, 2 and 3 years) and corresponding 95% CIs were calculated using the nonparametric Kaplan–Meier estimator^26^. Statistical comparison of the survival curves was performed using the log-rank test when stratification was performed on categorical variables.

The Cox PH model and RSF were both used to identify factors that affect CRC patient survival in our study. The well-known Cox PH model was used to estimate the effect of prognostic factors on survival time. The Multivariate Cox PH model with all the potential baseline predictors was estimated in order to compute the hazard ratio (HR) and their associated 95% CIs^21^. Baseline predictors were: sex, age, insurance, residency, delay to treatment, personal history, tumor site, stage, tumor differentiation, histological type, surgery, and MSI/MSS status.

Beyond the Kaplan–Meier estimator, the Cox PH model allows the inclusion of covariates, which is useful for refining the information on survival time. In this case, the statistical significance of the adjusted covariates on survival times is tested.

### Cox proportional hazard model

The Cox PH is a semi-parametric model with two components, one parametric related to predictor variables and another fully nonparametric related to the estimate of the survival function, which doesn’t make any assumptions about the underlying distribution of survival times.

In practice, the Cox model is specified by a hazard function:

$$h\left(t\right)={h}_{0}\left(t\right)exp\left({\beta }^{T}{X}_{i}\right)$$

where ${h}_{0}\left(t\right)$ is the risk function (i.e. instantaneous risk of death at inclusion). This risk function is modified by changes in survival time conditional on covariates ${X}_{i}={X}_{1},...,{X}_{p}$ which is a vector of covariates that do not depend on time, and ${\beta }_{i}={\beta }_{1},...,{\beta }_{p}$ is a vector of regression coefficients associated to ${X}_{i}$. The parameters of the vector ${\beta }_{i}$ are estimated by maximizing the partial true likelihood expressed as follows:

$$L\left(\beta \right)={\prod }_{i=1}^{m}\frac{exp\left({\beta }^{T}{X}_{i}\right)}{{\sum }_{j\in {R}_{i}}exp\left({\beta }^{T}{X}_{i}\right)}$$

where ${R}_{i}$ is the set of subjects at risk at time ${t}_{i}$, either ${y}_{i}\ge {t}_{i}$.

Several approaches can be used for model selection. A simple method is to estimate a univariate regression model, then a multivariate model with all predictors statistically significant (p-value > $\alpha$, $\alpha =0.05$). However, this univariate statistical significance filtering approach does not take into account interactions between covariates.

Recognizing the multitude of statistical tools available, we implemented a comprehensive regression modeling strategy to evaluate the robustness of the final Cox PH model fitted. This included diagnostic checks for the proportionality of hazards, evaluation of linearity for continuous variables, and investigation into potential outliers or influential cases.

It should be noted that, upon identifying deviations from the Cox model hypotheses, we did not employ typical modifications such as stratification or the inclusion of time-dependent covariates. This was a deliberate choice, aligning with our study's ultimate aim. Our primary objective was not to fine-tune the Cox PH model for optimal integrity and applicability, but rather to present an approach centered on the interpretability of a machine learning-based prognostic algorithm.

### Random survival forests

In order to identify the most influential predictors of survival outcome, we extended our data analysis by performing a RSF. The RSF is an extension of the classical random forest framework to right-censored observations^11,27, 29^.

The main advantage of this approach is that it does not require any restrictive assumptions on the distribution of the data, unlike the proportional hazard assumption for the Cox model^31^. As a first step, binary survival trees are developed using the bootstrap sampling procedure for all predictors included in the analysis, by recursive partitioning similar to CART^32^. Each bootstrap sample excludes about 37% of the out-of-bag (OOB) data used as an estimate of the predictive error. Then the log-rank test statistic is specified as the default dump rule for splitting survival trees^33^.

The final forest set is calculated by averaging the end node statistics using the boosted Nelson-Aalen and Kaplan–Meier estimators^29^.

### Variable importance

Another approach to covariate selection is the variable importance method (VIMP) based on permutation. With this method, the attributable prediction error of each predictor ${X}_{i}$ is calculated. This approach is defined by^34^ as the difference in model predictive performance between datasets with and without permuted values for the associated variable.

Permutation-based VIMP as implemented in RSF permutes the OOB data of a variable and compares its OOB prediction error with the original one. The intuition behind this method is that large importance values indicate variables with strong predictive potential^11,27, 28^.

### Partial dependence plots (PDP)

Futhermore, PDP were displayed to explore in depth the relationship between the estimated partial effect of a given predictor and survival rates^29^.

PDP can reveal the shape of the relationship between a covariate and the target variable. Its values are constructed by drawing a subset of patients at random and then predicting their survival with the random forest many times, while holding all the covariates constant except for the covariate for which we want to estimate its marginal effect. This gives risk curves for each individual in the subset, normalized by its mean to obtain a single risk curve. For categorical covariates, PDP gives the risk associated with a certain class given different values of the covariate^30^. Only the final set of strong predictors obtained by the VIMP procedure was considered in our interpretation.

### Predictive accuracy

Finally, we evaluated the predictive performance of our two models, Cox PH and RSF. The predictive performance was measured by two metrics, the Concordance Index (C-index) and the Brier Score (BS). The C-index is the frequency of concordant pairs among all pairs of subjects. It can be used to assess and compare the discriminative power of a risk prediction survival model^35^. A pair of patients $\left(i,j\right)$ is called concordant if the risk of the predicted event by the model is lower for the patient who experiences event at a later time point.

$$\text{C-index}=\frac{{\sum }_{i,j}{1}_{{T}_{j}<{T}_{i}}\cdot {1}_{{\eta }_{j}>{\eta }_{i}}\cdot {\delta }_{j}}{{\sum }_{i,j}{1}_{{T}_{j}<{T}_{i}}\cdot {\delta }_{j}}$$

with ${\eta }_{i}$ the risk score of a unit $i$ and ${1}_{{T}_{j}<{T}_{i}}=1$ if ${T}_{j}<{T}_{i}$ else 0 and ${1}_{{\eta }_{j}>{\eta }_{i}}=1$ if ${\eta }_{j}>{\eta }_{i}$ else 0. C-index takes values between 0 and 1, with 1 corresponding to the best discriminative power of the model.

The BS is used to evaluate the accuracy of a predicted survival function given a vector of time $t$. This is an improved version of the prediction error at the time point using inverse probability weighting of the censoring^36^.

$$BS\left(t\right)=\frac{1}{N}{\sum }_{i=1}^{N}\left[\frac{{\left(\widehat{S}\left(t\vee {z}_{i}\right)\right)}^{2}}{\widehat{G}\left({X}_{i}\right)}\cdot I\left({X}_{i}<t,{\delta }_{i}=1\right)+\frac{{\left(1-\widehat{S}\left(t\vee {z}_{i}\right)\right)}^{2}}{\widehat{G}\left(t\right)}\cdot I\left({X}_{i}\ge t\right)\right)$$

where $t$ is the time point at which BS is calculated, $N$ is the sample size, ${x}_{i}$ is the covariate corresponding to sample $i$; ${S}{\left(\cdot \right)}$ is the survival function predicted by the model and ${G}{\left(\cdot \right)}$ is the survival function corresponding to censoring. BS take values between 0 and 1, with 0 the best possible value corresponding to the best predictive accuracy of the model.

All statistical analyses were performed using R^37^. Specifically, Kaplan–Meier curves and cox models were computed using the survival package^1^. The finalfit package^38^ was used to display the univariate and multivariate cox regression tables. The survminer package^39,40^ was used to draw the KM curves. The missRanger package^24^ was used to impute missing values from the original data set. The RandomForestSRC package^41,42^ was used to build random survival forests, perform variable importance and display PDP.

## Results

A total of 346 patients were included for analysis. Details of demographic characteristics are described in Table 1. Overall, 181 were female (52.31%), with a mean age of 56.5 years (SD = 13.4).

**Table 1:** Socio-demographic characteristics of CRC patients.

	Female (N = 181)	Male (N = 165)	Overall (N = 346)
Age (years)			
Mean (SD)	56.5 (13.4)	58.3 (15.3)	57.3 (14.3)
Median [Min, Max]	57.0 [23.0, 82.0]	59.0 [19.0, 89.0]	58.0 [19.0, 89.0]
Insurance			
Yes	162 (89.5%)	151 (91.5%)	313 (90.5%)
No	19 (10.5%)	14 (8.5%)	33 (9.5%)
Residency			
Urban	146 (80.7%)	133 (80.6%)	279 (80.6%)
Rural	35 (19.3%)	32 (19.4%)	67 (19.4%)

Table 2 describes the clinical characteristics of CRC patients. There was almost an equal proportion of patients with colon (N = 186, 53%) and rectum (N = 160, 47%) cancers. The left colon was the most common tumor subsite (143, 41.3%), followed by the inferior (84, 24.3%) and middle rectum (58, 16.8%). More than half of the patients were at a distant stage at the time of inclusion (189, 54.6%). Furthermore, the predominant histological type was mucinous (313, 90.5%).

**Table 2:** Clinical characteristics of CRC patients.

	Colon (N = 186)	Rectum (N = 160)	Overall (N = 346)
Delay to start treatment (days)			
Mean (SD)	36.5 (51.7)	71.7 (72.9)	52.9 (64.8)
Median [Min, Max]	21.0 [0, 281]	53.0 [0, 503]	34.5 [0, 503]
Missing	6 (3.2%)	4 (2.5%)	10 (2.9%)
Personal history			
No	179 (96.2%)	157 (98.1%)	336 (97.1%)
Yes	7 (3.8%)	3 (1.9%)	10 (2.9%)
Tumor differentiation			
Moderately differentiated	66 (35.5%)	51 (31.9%)	117 (33.8%)
Undifferentiated	15 (8.1%)	11 (6.9%)	26 (7.5%)
Well differentiated	105 (56.5%)	98 (61.3%)	203 (58.7%)
Tumor subsite			
Inferior rectum	0 (0%)	84 (52.5%)	84 (24.3%)
Left colon	143 (76.9%)	0 (0%)	143 (41.3%)
Middle rectum	0 (0%)	58 (36.3%)	58 (16.8%)
Right colon	34 (18.3%)	0 (0%)	34 (9.8%)
Superior rectal	0 (0%)	18 (11.3%)	18 (5.2%)
Transverse colon	9 (4.8%)	0 (0%)	9 (2.6%)
Stage			
Local	52 (28.0%)	49 (30.6%)	101 (29.2%)
Regional	33 (17.7%)	23 (14.4%)	56 (16.2%)
Distant	101 (54.3%)	88 (55.0%)	189 (54.6%)
Histological type			
Mucinous	164 (88.2%)	148 (92.5%)	312 (90.2%)
Adenocarcinoma	17 (9.1%)	7 (4.4%)	24 (6.9%)
Signet ring cell	4 (2.2%)	5 (3.1%)	9 (2.6%)
Missing	1 (0.5%)	0 (0%)	1 (0.3%)
Surgery			
Yes	97 (52.2%)	88 (55.0%)	185 (53.5%)
No	89 (47.8%)	72 (45.0%)	161 (46.5%)
MSI/MSS Status			
No	154 (82.8%)	152 (95.0%)	306 (88.4%)
Yes	20 (10.8%)	4 (2.5%)	24 (6.9%)
Missing	12 (6.5%)	4 (2.5%)	16 (4.6%)
RAS mutated			
No	171 (91.9%)	153 (95.6%)	324 (93.6%)
Yes	7 (3.8%)	3 (1.9%)	10 (2.9%)
Missing	8 (4.3%)	4 (2.5%)	12 (3.5%)

Table 3 presents the overall survival rates at 1, 2 and 3 years, which are, respectively, 87% (SE = 0.02; CI-95% 0.84–0.91), 77% (SE = 0.02; CI-95% 0.73–0.82) and 60% (SE = 0.03; CI-95% 0.54–0.66).

**Table 3:** Overall survival rates at 1 year, 2 years and 3 years.

Year	Survival rates	SE	95% CI-lower	95% CI-upper
1 year	0.87	0.02	0.84	0.91
2 years	0.77	0.02	0.73	0.82
3 years	0.60	0.03	0.54	0.66

**Figure 1:**
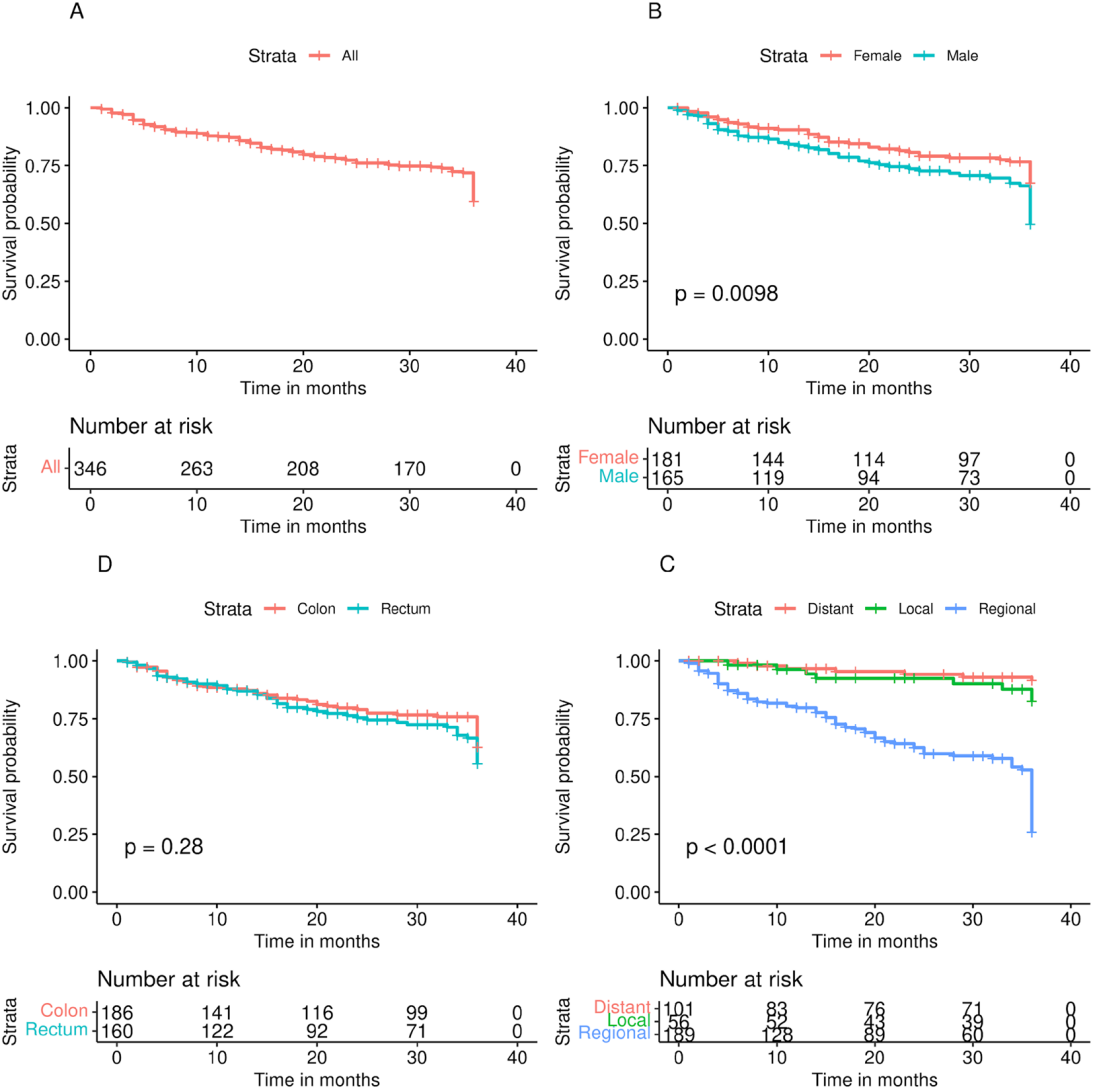
Kaplan-Meir survival curves.

The difference in the survival curve was statistically significant by sex (log-rank test, *p* < 0.0098). The difference was also statistically significant when the survival curve was stratified by stage (log-rank test, *p* = 0.0001). However, no statistical significant difference was observed when stratifying by site (log-rank test, *p* = 0.28) (Fig. 1).

Only MSI/MSS status, Delay and histological type had missing values, respectively, 4.6%, 2.89% and 0.28%.

### Cox proportional hazard models

The reuslts of the multivariate Cox PH model illustrate significant disparities in mortality risks associated with surgical intervention, cancer stage, insurance status, residential location, and tumor location. Specifically, patients who did not have surgery were 3.21 times more likely to die (HR 3.21; CI 1.83–5.63; *p* < 0.001) than those who did, and those with a distant stage were 6.64 times more likely to die (HR 6.64; CI 2.80–15.72; *p* < 0.001). Patients with no health insurance had 2.85 times a higher risk of mortality (HR 2.85; CI 1.63–4.98; *p* < 0.001) than patients with health insurance. Similarly, patients living in rural areas had a 1.88 times greater risk of death (HR 1.88; CI 1.18–2.98; *p* < 0.001) compared to those living in urban areas. Besides, patients with a tumor in the rectum side had a 1.86 times greater risk of death (CI 1.21; 2.88; *p* = 0.005) than patients with a tumor in the colon side (Table 4).

**Table 4:** Cox regression models.

Label	Levels	All	HR (Complete Cases)	HR (missRanger)	HR (mice RF)
Sex	Female	181 (52.3)	–	–	–
	Male	165 (47.7)	1.73 (1.13–2.64, * p* = 0.012)	1.46 (0.97–2.19, * p* = 0.067)	1.47 (0.97–2.23, * p* = 0.066)
Age	Mean (SD)	57.3 (14.3)	1.02 (1.00–1.03, * p* = 0.044)	1.02 (1.00–1.03, * p* = 0.032)	1.02 (1.00–1.03, * p* = 0.033)
Delay	Mean (SD)	52.9 (64.8)	1.00 (0.99–1.00, * p* = 0.193)	1.00 (1.00–1.00, * p* = 0.237)	1.00 (0.99–1.00, * p* = 0.187)
Insurance	No	33 (9.5)	–	–	–
	Yes	313 (90.5)	0.38 (0.21–0.69, * p* = 0.002)	0.35 (0.20–0.62, * p* < 0.001)	0.35 (0.20–0.62, * p* < 0.001)
Residency	Rural	67 (19.4)	–	–	–
	Urban	279 (80.6)	0.44 (0.26–0.73, * p* = 0.002)	0.53 (0.33–0.84, * p* = 0.007)	0.53 (0.33–0.85, * p* = 0.009)
Personal history	No	336 (97.1)	–	–	–
	Yes	10 (2.9)	1.07 (0.25–4.54, * p* = 0.930)	1.10 (0.26–4.66, * p* = 0.894)	1.09 (0.25–4.70, * p* = 0.909)
Site	Colon	186 (53.8)	–	–	–
	Rectum	160 (46.2)	1.89 (1.19–3.01, * p* = 0.007)	1.84 (1.19–2.84, * p* = 0.006)	1.86 (1.19–2.89, * p* = 0.007)
Stage	Distant	189 (54.6)	–	–	–
	Local	101 (29.2)	0.16 (0.07–0.39, * p* < 0.001)	0.15 (0.06–0.36, * p* < 0.001)	0.15 (0.06–0.36, * p* < 0.001)
	Regional	56 (16.2)	0.36 (0.15–0.85, * p* = 0.020)	0.32 (0.13–0.74, * p* = 0.008)	0.32 (0.13–0.76, * p* = 0.010)
Tumor differentiation	Undifferentiated	117 (33.8)	–	–	–
	Moderately differentiated	26 (7.5)	1.79 (0.68–4.74, * p* = 0.238)	1.67 (0.69–4.07, * p* = 0.259)	1.65 (0.67–4.06, * p* = 0.274)
	Well differentiated	203 (58.7)	0.87 (0.55–1.39, * p* = 0.568)	0.90 (0.57–1.40, * p* = 0.630)	0.89 (0.56–1.40, * p* = 0.609)
Histological type	Mucinous	312 (90.4)	–	–	–
	Adenocarcinoma	24 (7.0)	1.03 (0.37–2.83, * p* = 0.955)	1.11 (0.44–2.77, * p* = 0.830)	1.11 (0.44–2.82, * p* = 0.821)
	Signet ring cell	9 (2.6)	2.08 (0.48–9.12, * p* = 0.330)	2.16 (0.52–8.93, * p* = 0.288)	2.20 (0.52–9.29, * p* = 0.280)
Surgery	No	161 (46.5)	–	–	–
	Yes	185 (53.5)	0.29 (0.16–0.52, * p* < 0.001)	0.31 (0.18–0.55, * p* < 0.001)	0.31 (0.18–0.55, * p* < 0.001)
MSI.MSS status	No	306 (92.7)	–	–	–
	Yes	24 (7.3)	0.98 (0.40–2.41, * p* = 0.958)	1.06 (0.43–2.60, * p* = 0.903)	1.03 (0.42–2.55, * p* = 0.952)
Ras mutated	No	324 (97.0)	–	–	–
	Yes	10 (3.0)	0.92 (0.36–2.33, * p* = 0.855)	0.83 (0.33–2.09, * p* = 0.694)	0.78 (0.30–2.05, * p* = 0.616)

After the sensitivity analysis performed by RSF, we decided to keep all predictors in the multivariate Cox PH model in order to preserve the interaction between covariates, while interpreting covariates that have a statistically significant effect in the Cox model, and the presence of their effect has been confirmed by the RSF.

### RSF variable importance

The RSF was fitted using the same covariates as in the Cox PH models. A variable importance permutation-based approach was used to identify the most important prognostic factors related to the overall survival of our group of patients, and confidence intervals were calculated using subsampling.

**Figure 2:**
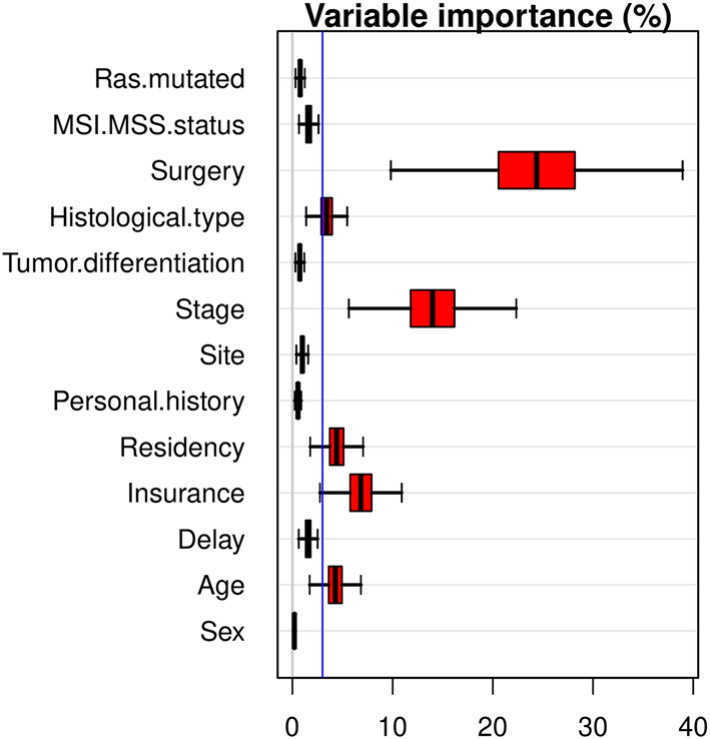
Variable importance of random survival forest through permutation.

The variable importance obtained from RSF strengthens that surgery, stage, insurance, residency, and age were the most important prognostic factors (Fig. 2).

These results are in agreement with the results obtained from fitting the multivariate Cox PH model (Table 4), as far as statistical significant effects are concerned.

**Figure 3:**
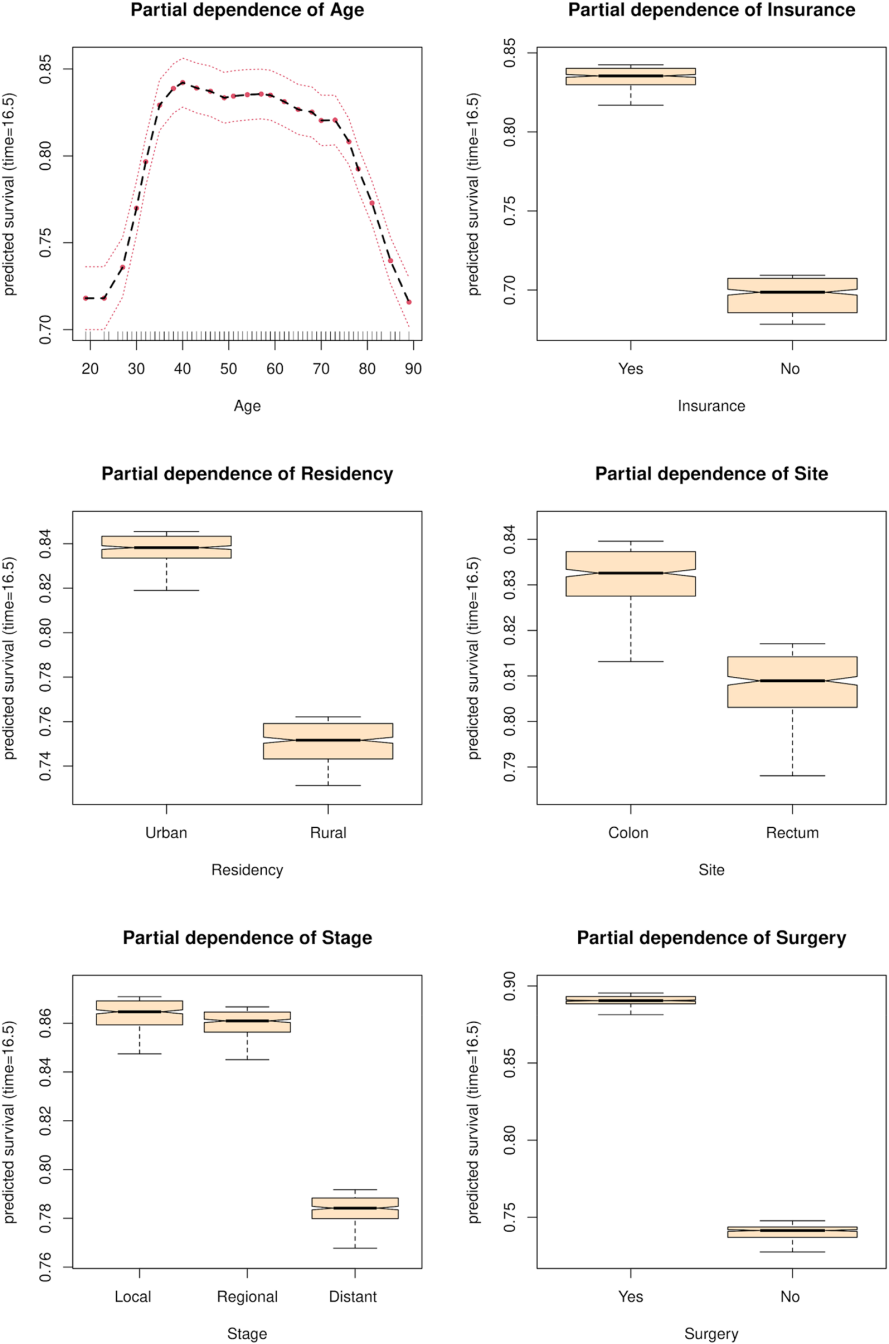
Partial dependence plots.

Figure 3 depicts PDP. For age, PDP shows that the survival rate increases from 18 to 30 years, decreases very slowly between 30 and 75 years, and drops after that age. For insurance, we observed a positive effect for the ‘Yes’ modality compared to the ‘No’ modality on the survival rate, with a relative difference of almost 15 percentage points. For residency, we observed a positive effect on the survival rate for the ‘Urban’ modality compared to the ‘Rural’ modality, with a relative difference of almost 9 percentage points. For the site, a positive effect on the survival rate for the ‘Colon’ modality compared to the ‘Rectum’ modality was shown, with a relative difference of almost 3 percentage points. Furthermore, the ‘Distant’ stage had a negative effect on the survival rate when compared to the ‘Local’ stage, with a relative difference of nearly 7 percentage points. For surgery, the ‘Yes’ modality had a positive effect on the survival rate compared to the ‘No’ modality, with a relative difference of almost 15 percentage points.

### Predictive performance

**Figure 4:**
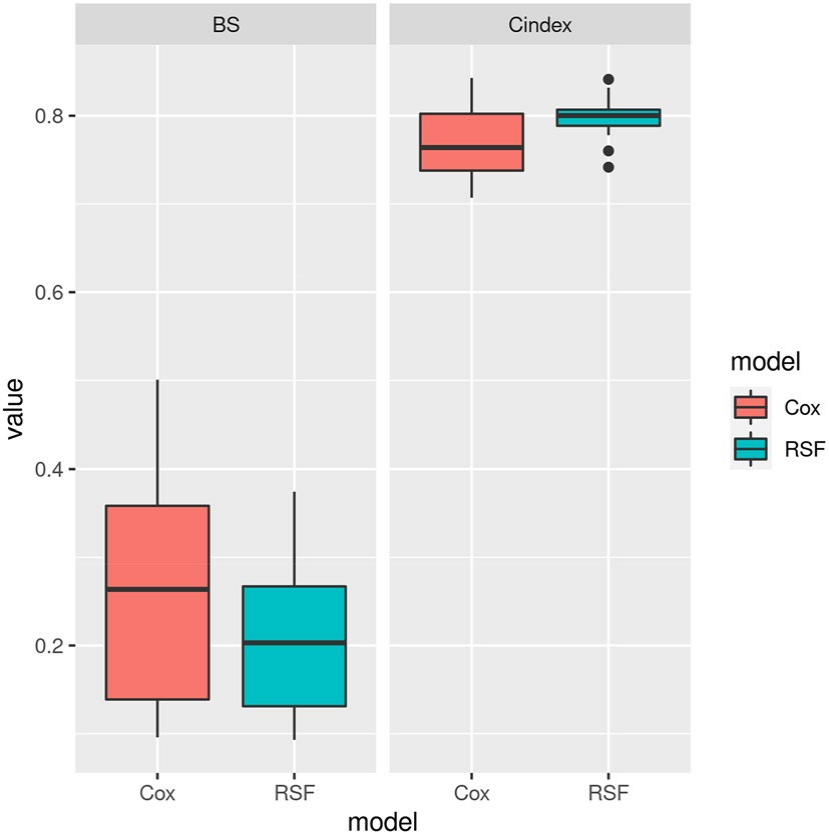
Predictive performance using C-index and BS.

The predictive performance of the Cox PH and RSF was evaluated using the C-index and the Integrated BS. The discriminative capacity of the Cox PH and RSF was, respectively, 0.771 and 0.798 for the C-index. while the accuracy of the Cox PH and RSF were, respectively, 0.257 and 0.207 for the BS. This shows that RSF had both a better discriminative capaciy and predictive accuracy (Fig. 4).

## Discussion

CRC is one of the most common cancers diagnosed globally. However, due to earlier detection and more effective treatment, high-income countries have seen a significant decrease in CRC mortality over the past decades. Personalizing treatment, focusing on high-risk patients, and improving access to health-care systems are critical to achieving the best possible health outcome.

In Morocco, there are two Population-Based Cancer Registries (PBCR): one in Casablanca that covers about 12% of the national population (36 million people) and has 38 data sources, and another in Rabat that covers about 21% of the national population (642,000 people) and has 65 data sources^43,44^. But as it is known, PBCR make a trade-off between exhaustivity in terms of incidence and availability of variables about patients (i.e., prognostic factors). Therefore, the study of factors influencing survival times is a difficult exercise with this type of data. Nevertheless, we have attempted for the first time in Morocco to study the survival of CRC and associate it with both socio-demographic factors and histopathological characteristics.

We found only one Moroccan study in the literature on the effect of predictive factors, particularly surgery, on survival of patients with mid and low rectal aenocarcinoma^45^. The overall survival rate at 3 years was found to be 82%. However, this survival rate should be viewed with caution due to the small sample size (81 patients) but also the specificity of the patients who had the same histological type.

The overall survival at 3 years observed in our cohort was 0.6 [SE = 0.03; CI 95% 0.54–0.66], which is close to the results found in the literature. In a meta-analysis conducted on individual studies done in Iran, the pooled 3 years survival rate estimated by a random effect model was 0.64 [CI 95% 0.59–0.7]^12^. This result is very close to the rate found by^46^, which is 70.67 [CI 95% 66.4–74.93] in EMRO countries.

The literature on prognostic factors for CRC is extremely rich^47,48^. Regarding age, our results reveal a high-risk period after 72 years and a low-risk period before 30 years (Fig. 3). This result is interesting because the majority of studies on CRC predictive factors use age as a categorical variable through-age groups, thus losing an estimate of their prognostic effect at age time points^47,49,50^.

We also identified factors that are considered to be barriers to health care seeking for CRC patients. Our results reveal that health insurance, pathological stage, and surgery were the main prognostic factors. Age, residency, and site also have a significant impact on the predicted survival rate.

In fact, the health coverage of patients is a condition to their regular access to care and thus have an important impact on their survival probability after diagnosis. The influence of financial barriers affects perceptions of severity, importance, and attribution of symptoms. When patients believe they cannot afford treatment, they may be more likely to downplay the severity of their symptoms^51^.

Patients must first detect and interpret CRC symptoms as requiring medical attention before approaching the health system. When this does not happen, it is referred to as an apparaisal delay^51^ Assessment delay is defined as the time between when patients first detects their symptoms and when they first disclose them to a health professional. This delay is even more important when patients do not have health insurance and access to care is difficult.

In addition, other factors such as geographical distance and unfavourable economic conditions may also delay diagnosis. In a study done on a large panel of patients, the risk of death increased significantly beyond 31 days from diagnosis to treatment interval^52^. We found that living in a rural area has a negative effect on CRC survival. This is closely related to the large distance between the health care centers and the places of residence in rural areas.

Morever, a prevention strategy based on the individual risk of CRC should be systematically adopted by health professionals^53^. Primary prevention for the general population is important. However, in order to be more effective, it should be targeted at the high-risk population^54^. Our results show that people aged 30 years or older, living in rural areas, without health insurance, at a distant stage and who have not had surgery, constitute a subgroup of patients with poor prognonsis. Therefore, screening programs should be offered systematically to those patients.

The good predictive performance of RSF is an interesting result, especially in the context of predicting prognostic factors. RSF are flexible and have no assumptions about the data in hand. Even though they are considered black-box models, the methodological development of interpretability techniques has made their results more intelligible. Therefore, they are a good alternative model for analyzing survival data for cancer studies^55,56^.

The RSF offers a fresh perspective and methodology in survival analysis, leveraging the power of ensemble machine learning. Unlike the classical Cox PH model, which assumes proportional hazards and relies heavily on predefined assumptions about variable relationships, RSF offers a more flexible approach. It inherently accounts for interactions between variables, non-linear effects, and complex relationships within the data without needing explicit specification. This adaptability and robustness make RSF particularly advantageous in handling intricate datasets where traditional methods might fall short. In this regard, the RSF model, being a tree-based ensemble non-parametric algorithm, can address the limitations of the Cox model while also identifying and ranking the most important variables affecting survival time^57^.

It is necessary to combine several methods to ensure consistency of results. This is the case in our study. We combined conventional methods such as the Kaplan–Meier estimator and the Cox PH model with the RSF method and the PDP interpretation approach. The agreement between the methods used confirms the low degree of sensitivity of our results^15^. Even though in our case, the assumptions of the Cox model were no met (see supplementary_file.docx). As a result, we cannot draw definitive conclusions based on the Cox model in our case; however, we utilized it as a reference, a baseline model, in order to shed light on the value of a data-driven approach. This comparison allows us to underscore the potential enhancements a machine learning methodology can offer, especially when handling the complexity and volume of data typically encountered in electronic health records (EHR). It also provides a framework for evaluating the degree to which advanced analytical techniques can complement or surpass traditional statistical models in the realm of prognostic modeling.

This study acknowledges certain limitations that warrant consideration. Primarily, the assumptions of the Cox PH model, central to our analysis, were not fully met, limiting the validity of the Cox model results. Additionally, the absence of a preliminary simulation study to validate the Cox PH model and (RSF methods may limit the generalizability of our findings^58^. Furthermore, challenges related to the sample size and potential selection bias, inherent in observational studies using EHR also impact our study. These factors, could influence the representativeness of our results, necessitating a cautions interpretation and highlighting areas for future research improvement. Our study is the first in Morocco based on an innovative and robust statistical approach. Thus, the sample studied was subjected to an important data quality verification work. Even if our data come from patient files, they have been completed if necessary by an actif follow-up reflecting more precision for the endpoint.

It is clear that our cohort recruited at Hassan II University Hospital cannot claim to be representative of the Moroccan population, but this will be improved in our future studies, where we intend to design a multicenter study to reflect the profile of Moroccan CRC patients as much as possible. Furthermore, adding data on the genetic profile of the included patients is an excellent way o obtain very revealing results^59^.

Also, a clinical decision support platform for CRC is needed in order to make clinical information easily usable by practitioners. Unfortunately, we are limited by the retrospective nature of our study. Ideally, the physician should have access to such a tool at each suspicious consultation to predict a risk score of developing CRC in order to perform real-time clinical prevention. Besides, the risk of information bias is not totally discarded, especially for disadvantaged patients with difficulties accessing care.

## Conclusion

Our research has highlighted that the RSF approach demonstrates better performance in scenarios where the assumptions of the Cox PH model are not valid. Thus, it’s more appropriate to view RSF  as a better option when the Cox PH models’s assumptions are chellenged, rather than as universally superior. This highlight the significance of choosing the right method based on the particular conditions and assumptions of each study.

Utilizing data-driven techniques not only streamlines the model-building process for researchers but also paves the way for the discovery of new predictors with substantial epidemiological importance. Intriguingly, these methods are not tethered to specific diseases, making them adaptable for studying conditions whose origins are not yet fully understood. Managing and addressing the multifaceted challenges of CRC requires more than just traditional therapeutic interventions. There's an imperative to embrace predictive and personalized medical strategies at policy-making levels for comprehensive and effective disease control.

To enhance the efficacy of such strategies, we must emphasize the preventable aspects of CRC and prioritize ensuring healthcare accessibility for the most susceptible sections of the population. Expanding health insurance coverage emerges as a pressing national requirement. Universal health coverage can significantly mitigate cancer risks by fostering an environment conducive to early detection and minimizing the financial burdens primarily affecting those with limited means.

From a methodological perspective, machine learning offers a transformative potential in prognostic studies. It facilitates the identification of complex relationships between predictors and outcomes, leading to intriguing epidemiological findings. In contrast, traditional methods often operate within deterministic boundaries.

In line with this research, the development of a clinical decision support system would be immensely beneficial. Such a system would empower clinicians to formulate prognoses in a more personalized and dynamic manner, aligning with the individualized needs of patients. By harnessing the insights from machine learning and predictive analytics, clinicians can make informed decisions, optimizing treatment pathways and potentially improving patient outcomes. This shift towards a data-driven, patient-centric approach is the future of medical prognosis and can redefine how we address the challenges of diseases like CRC in real-time clinical settings.

### Supplementary Information

Supplementary Figures.

## Data Availability

The dataset analysed during the current study is available in the figshare repository, accessible via this link: https://figshare.com/articles/dataset/dataset_CRC_Fes_csv/21821439.

## References

[1] 504-Morocco-fact-sheets.pdf. https://gco.iarc.fr/today/data/factsheets/populations/504-morocco-fact-sheets.pdf. Accessed August 2022.

[2] Bai J, Zhang X, Xiang ZX, Zhong PY, Xiong B (2020). Identification of prognostic immune-related signature predicting the overall survival for colorectal cancer. Eur. Rev. Med. Pharmacol. Sci..

[3] Lee Y-H, Kung P-T, Wang Y-H, Kuo W-Y, Kao S-L, Tsai W-C (2019). Effect of length of time from diagnosis to treatment on colorectal cancer survival: A population-based study. PLoS ONE.

[4] Mayer, M. Package ‘missRanger’. *R Package* (2019).

[5] Berraho M, Obtel M, Bendahhou K, Zidouh A, Errihani H, Benider A, Nejjari C (2012). Sociodemographic factors and delay in the diagnosis of cervical cancer in Morocco. Pan Afr. Med. J..

[6] Siminoff L, Thomson M, Dumenci L (2014). Factors associated with delayed patient appraisal of colorectal cancer symptoms. Psycho-Oncology.

[7] Stekhoven DJ, Bühlmann P (2012). MissForest—non-parametric missing value imputation for mixed-type data. Bioinformatics.

[8] Becker G (2004). Deadly inequality in the health care ‘safety net’: Uninsured ethnic minorities’ struggle to live with life-threatening illnesses. Med. Anthropol. Q..

[9] Chow Z, Osterhaus P, Huang B, Chen Q, Schoenberg N, Mark Dignan B, Evers M, Bhakta A (2021). Factors contributing to delay in specialist care after colorectal cancer diagnosis in Kentucky. J. Surg. Res..

[10] Courtney RJ, Paul CL, Sanson-Fisher RW, Macrae F, Attia J, McEvoy M (2012). Current state of medical-advice-seeking behaviour for symptoms of colorectal cancer: Determinants of failure and delay in medical consultation. Colorectal Dis..

[11] Breiman L (2001). Random forests. Mach. Learn..

[12] Magaji BA, Moy FM, Roslani AC, Law CW (2017). Survival rates and predictors of survival among colorectal cancer patients in a Malaysian tertiary hospital. BMC Cancer.

[13] Nikbakht HA, Hassanipour S, Shojaie L, Vali M, Ghaffari-fam S, Ghelichi-ghojogh M, Maleki Z (2020). Survival rate of colorectal cancer in eastern mediterranean region countries: A systematic review and meta-analysis. Cancer Control.

[14] De Rosa M (2016). The biological complexity of colorectal cancer: Insights into biomarkers for early detection and personalized care. Therapeutic Adv. Gastroenterol..

[15] Compton CC (2018). Precision medicine core: Progress in prognostication—Populations to patients. Ann. Surg. Oncol..

[16] Steele AJ, Denaxas SC, Shah AD, Hemingway H, Luscombe NM (2018). Machine learning models in electronic health records can outperform conventional survival models for predicting patient mortality in coronary artery disease. PLoS One.

[17] Collins, A.R. & Yao, Y. Machine learning approaches: Data integration for disease prediction and prognosis (2018).

[18] van der Schaar, M. & Hemingway, H. Machine learning in prognosis research. Prognosis Res. Health Care (2019).

[19] Weathers, B. & Cutler, R. Comparision of survival curves between cox proportional hazards, random forests, and conditional inference forests in survival analysis (2017).

[20] Cruz JA, Wishart DS (2006). Applications of machine learning in cancer prediction and prognosis. Cancer Inform..

[21] Cox DR (1972). Regression models and life-tables. J. R. Stat. Soc. Ser. B (Methodol.).

[22] Herring AH (2004). Non-ignorable missing covariate data in survival analysis: A case-study of an International Breast Cancer Study Group trial. J. R. Stat. Soc. Ser. C (Appl. Stat.).

[23] Apte M (2011). Using electronically available inpatient hospital data for research. Clin. Transl. Sci..

[24] Miao, F., Cai, Y.-P., Zhang, Y.-X., Li, Y. & Zhang, Y.-T. Risk prediction of one-year mortality in patients with cardiac arrhythmias using random survival forest. *Comput. Math. Methods Med.* 2015 (2015). 10.1155/2015/303250 PMC4562335 26379761

[25] Tazi MA, Er-Raki A, Benjaafar N (2013). Cancer Incidence in Rabat, Morocco: 2006–2008. Ecancermedicalscience.

[26] Kaplan EL, Meier P (1958). Nonparametric estimation from incomplete observations. J. Am. Stat. Assoc..

[27] Breiman L (1996). Bagging predictors. Mach. Learn..

[28] Ishwaran H, Min Lu (2019). Standard errors and confidence intervals for variable importance in random forest regression, classification, and survival. Stat. Med..

[29] Ishwaran H, Kogalur UB, Blackstone EH, Lauer MS (2008). Random survival forests. Ann. Appl. Stat..

[30] Ishwaran, H., Lu, M. & Kogalur, U. B. randomForestSRC: Partial Plots Vignette. http://randomforestsrc.org/articles/partial.html (2021).

[31] Murphy N, Moreno V, Hughes DJ, Vodicka L, Vodicka P, Aglago EK, Gunter MJ, Jenab M (2019). Lifestyle and dietary environmental factors in colorectal cancer susceptibility. Mol. Asp. Med..

[32] Crawford SL (1989). Extensions to the CART algorithm. Int. J. Man-Mach. Stud..

[33] Ciampi A, Negassa A, Lou Z (1995). Tree-structured prediction for censored survival data and the cox model. J. Clin. Epidemiol..

[34] Fisher A, Rudin C, Dominici F (2019). All models are wrong, but many are useful: Learning a variable’s importance by studying an entire class of prediction models simultaneously. J. Mach. Learn. Res..

[35] Harrell FE, Califf RM, Pryor DB, Lee KL, Rosati RA (1982). Evaluating the yield of medical tests. JAMA.

[36] Graf E, Schmoor C, Sauerbrei W, Schumacher M (1999). Assessment and comparison of prognostic classification schemes for survival data. Stat. Med..

[37] Roncucci L, Mariani F (2015). Prevention of colorectal cancer: How many tools do we have in our basket?. Eur. J. Internal Med..

[38] Harrison, E., Drake, T., Ots, R. & Harrison, M. E. Package ‘Finalfit.’ (2020).

[39] Kassambara, A., Kosinski, M., Biecek, P. & Fabian, S. Package ‘Survminer’. *Drawing Survival Curves Using “Ggplot2”(R Package Version 03 1)* (2017).

[40] Lee CH, Cheng SC, Tung HY, Chang SC, Ching CY, Shu Fen Wu (2018). The risk factors affecting survival in colorectal cancer in Taiwan. Iran. J. Public Health.

[41] Ishwaran H, Kogalur UB (2022). Package ‘randomForestSRC’. Breast.

[42] Ishwaran, H., Lauer, M. S., Blackstone, E. H., Lu, M. & Kogalur, U. B. randomForestSRC: Random Survival Forests Vignette. http://randomforestsrc.org/articles/survival.html (2021).

[43] Bouchbika Z, Haddad H, Benchakroun N, Kotbi S, Megrini A, Bourezgui H, Sahraoui S, Corbex M, Harif M, Benider A (2014). Cancer incidence in Morocco: Report from Casablanca Registry 2005–2007. Pan Afr. Med. J..

[44] Volovici V (2022). Steps to avoid overuse and misuse of machine learning in clinical research. Nat. Med..

[45] Essangri H, Majbar MA, Benkabbou A, Amrani L, Belkhadir Z, Ghennam A, Al Ahmadi B, Bougtab A, Mohsine R, Souadka A (2019). Predictive factors of oncological and survival outcome of surgery on mid and low rectal adenocarcinoma in Morocco: Single center study. J. Med. Surg. Res..

[46] Omurlu IK, Ture M, Tokatli F (2009). The comparisons of random survival forests and cox regression analysis with simulation and an application related to breast cancer. Expert Syst. Appl..

[47] Atinafu BT, Bulti FA, Demelew TM (2020). Survival status and predictors of mortality among colorectal cancer patients in Tikur Anbessa specialized hospital, Addis Ababa, Ethiopia: A retrospective followup study. J. Cancer Prevent..

[48] R Core Team. *R: A Language and Environment for Statistical Computing*. R Foundation for Statistical Computing. https://www.R-project.org/ (2021)

[49] Lee C-H, Cheng S-C, Tung H-Y, Chang S-C, Ching C-Y, Wu S-F (2018). The risk factors affecting survival in colorectal cancer in Taiwan. Iran. J. Public Health.

[50] Farhat W, Azzaza M, Mizouni A, Ammar H, ben Ltaifa M, Lagha S, Kahloul M, Gupta R, Ben Mabrouk M, Ben Ali A (2019). Factors predicting recurrence after curative resection for rectal cancer: A 16-year study. World J. Surg. Oncol..

[51] Sung H, Ferlay J, Siegel RL, Laversanne M, Soerjomataram I, Jemal A, Bray F (2021). Global cancer statistics 2020: GLOBOCAN estimates of incidence and mortality worldwide for 36 cancers in 185 countries. CA A Cancer J. Clin..

[52] Louppe, G., Wehenkel, L., Sutera, A. & Geurts, P. Understanding variable importances in forests of randomized trees. In *Advances in Neural Information Processing Systems* 26 (2013).

[53] Sharma R (2020). An examination of colorectal cancer burden by socioeconomic status: Evidence from GLOBOCAN 2018. EPMA J..

[54] Giovannucci E (2002). Modifiable risk factors for colon cancer. Gastroenterol. Clin..

[55] Mogensen UB, Ishwaran H, Gerds TA (2012). Evaluating random forests for survival analysis using prediction error curves. J. Stat. Softw..

[56] Ortiz-Ortiz KJ, Ríos-Motta R, Marín-Centeno H, Cruz-Correa M, Ortiz AP (2016). Factors associated with late stage at diagnosis among Puerto Rico’s government health plan colorectal cancer patients: A cross-sectional study. BMC Health Serv. Res..

[57] Farhadian M, DehdarKarsidani S, Mozayanimonfared A, Mahjub H (2021). Risk factors associated with major adverse cardiac and cerebrovascular events following percutaneous coronary intervention: A 10-year follow-up comparing random survival forest and Cox proportional-hazards model. BMC Cardiovasc. Disord..

[58] Smith H, Sweeting M, Morris T, Crowther MJ (2022). A scoping methodological review of simulation studies comparing statistical and machine learning approaches to risk prediction for time-to-event data. Diagn. Prognostic Res..

[59] Carr PR, Weigl K, Jansen L, Walter V, Erben V, Chang-Claude J, Brenner H, Hoffmeister M (2018). Healthy lifestyle factors associated with lower risk of colorectal cancer irrespective of genetic risk. Gastroenterology.

