# Author Correction: Risk factors affecting patients survival with colorectal cancer in Morocco: survival analysis using an interpretable machine learning approach

**DOI:** 10.1038/s41598-024-60557-x

**Published:** 2024-05-01

**Authors:** Imad El Badisy, Zineb BenBrahim, Mohamed Khalis, Soukaina Elansari, Youssef ElHitmi, Fouad Abbass, Nawfal Mellas, Karima EL Rhazi

**Affiliations:** 1 Mohammed VI Center for Research and Innovation, Rabat, Morocco; 2 International School of Public Health, Mohammed VI University of Sciences and Health, Casablanca, Morocco; 3 INSERM, IRD, SESSTIM, Sciences Economiques & Sociales de la Santé & Traitement de l’Information Médicale, Aix Marseille Univ, Marseille, France; 4 Faculty of Medicine, Pharmacy & Dental Medicine, Sidi Mohamed Ben Abdillah University, Fez, Morocco; 5 Department of Oncology, University Hospital Hassan II, Sidi Mohamed Ben Abdellah University, Fez, Morocco; 6 Higher Institute of Nursing Professions and Technical Health, Rabat, Morocco; 7 Laboratory of Biostatistics, Clinical, and Epidemiological Research, Faculty of Medicine and Pharmacy, Department of Public Health, Mohamed V University, Rabat, Morocco; 8 Laboratory of Epidemiology and Research in Health Sciences, Department of Epidemiology and Public Health, Faculty of Medicine of Fez, Sidi Mohamed Ben Abdillah University, Fez, Morocco

Correction to: *Scientific Reports* 10.1038/s41598-024-51304-3, published online 12 February 2024

The original version of this Article contained an error in the spelling of the author Zineb BenBrahim which was incorrectly given as Zineb Ben Brahim.

In addition, Zineb BenBrahim was incorrectly affiliated with ‘Department of Oncology, University Hospital Hassan II, Sidi Mohamed Ben Abdellah University, Fez, Morocco’. The correct affiliation is listed below.

Faculty of Medicine, Pharmacy & Dental Medicine, Sidi Mohamed Ben Abdillah University, Fez, Morocco.

The original Article has been corrected.

