# Supplementary material for: Risk factors affecting patients survival with colorectal cancer in Morocco: survival analysis using an interpretable machine learning approach
Source: Sci Rep. 2024 Feb 12;14:3556. doi: 10.1038/s41598-024-51304-3 (PMC10861582; doi:10.1038/s41598-024-51304-3)
Supplement: Supplementary file 1 — Supplementary Figures. [file 41598_2024_51304_MOESM1_ESM.docx]

**Additional figures**

**Figure S1: Martingale residuals for non-linearity detection**


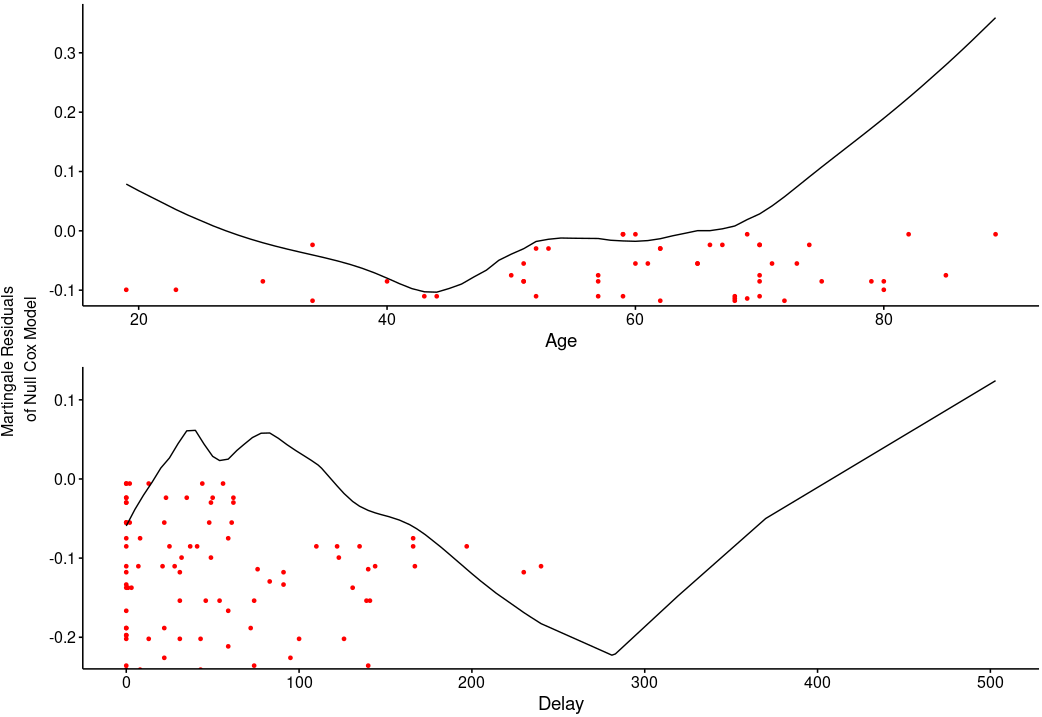


Visualizing Martingale Residuals for detecting non-linearity in age and delay variables.

**Figure S2: Schoenfeld residuals plots for checking proportional hazards assumption**


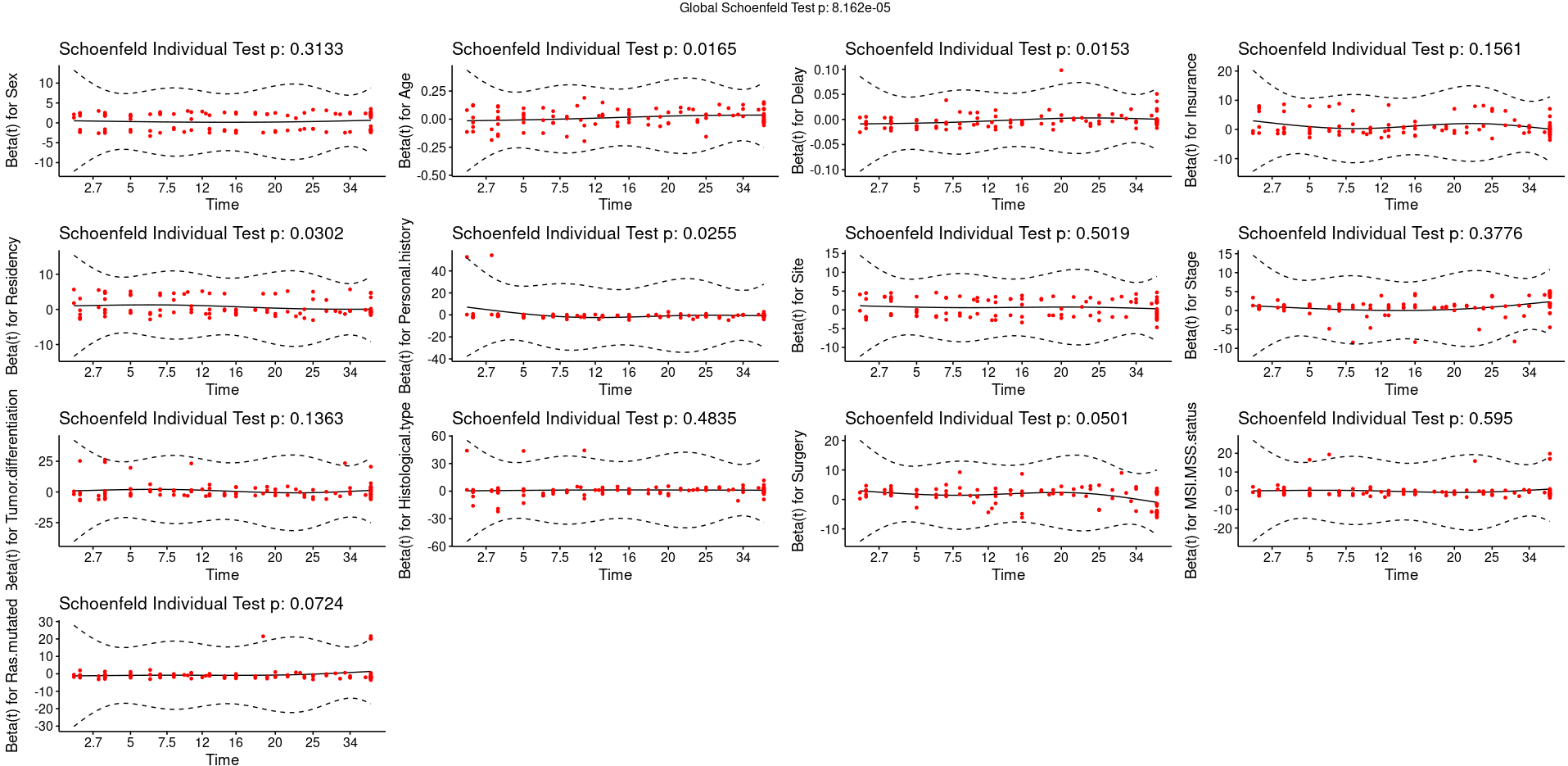
Evaluating Proportional Hazards using Schoenfeld Residuals plots for each covariate.

**Figure S3: Influential cases**


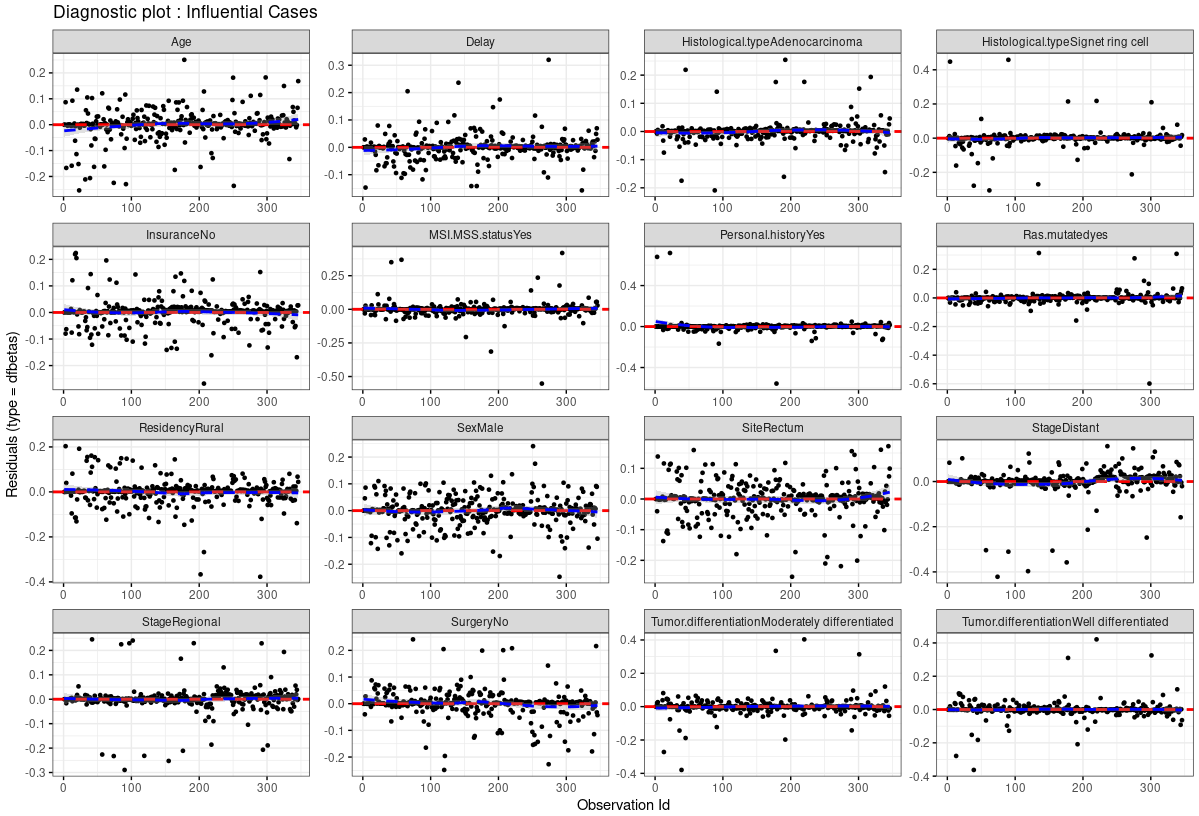


A plot of influential cases in the analysis.

**Figure S4: Comparison of survival curves**


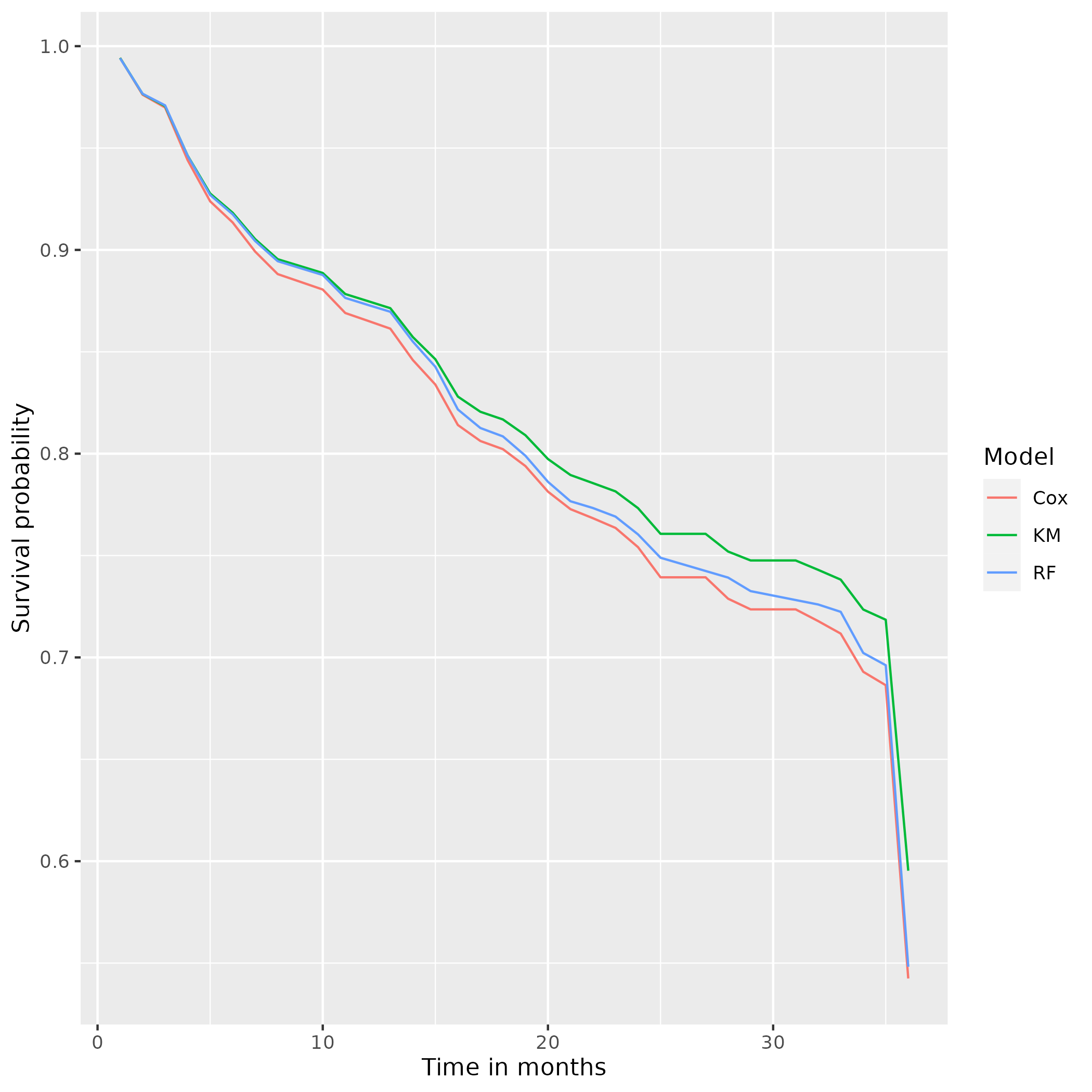


Overlay of survival curves, a side-by-side comparison on a single plot.
